# Antioxidant and antimicrobial activities of different enzymatic hydrolysates from desalted duck egg white

**DOI:** 10.5713/ajas.19.0361

**Published:** 2019-12-24

**Authors:** Rommanee Thammasena, Deng Cheng Liu

**Affiliations:** 1 Department of Animal Science, National Chung Hsing University, Taichung 40227, Taiwan

**Keywords:** Desalted Duck Egg White, Hydrolysate, Antioxidant Activity, Antimicrobial Activity

## Abstract

**Objective:**

The objective of this study was to look for optimal preparation of hydrolysates of desalted duck egg white powder (DDEWP) by the three different proteases and to investigate their antioxidant and antimicrobial properties.

**Methods:**

DDEWP was hydrolyzed by three proteases, including pepsin (PEP), *Bacillus* spp. (BA) and natokinase (NAT) with three different enzyme concentrations (0.1%, 0.3%, and 0.5%), individually. The important key hydrolysis parameters such as hydrolysis degree, yield, antioxidant and antimicrobial activity were evaluated in this experiment.

**Results:**

The results showed that the degree of hydrolysis (DH) of all treatments increased with increasing hydrolysis time and protease concentrations. The antioxidant and antimicrobial activities of the hydrolysates were affected by type and concentration of protease as well as hydrolysis time. Hydrolysis of PEP significantly (p<0.05) obtained the highest yield of hydrolysates, however, both of BA and NAT showed substantially lower DH values and still did not exceed 5% by the end of hydrolysis. Among the different hydrolysates, PEP exhibited significantly higher 2, 2–diphenyl-1-picrylhydrazyl (DPPH) radical scavenging activity than BA and NAT. All DDEWP hydrolysates from PEP had low ferrous ion chelating activity (<37%) that was significantly lower than that of NAT (>37% to 92%) and BA (30% to 79%). Besides, DDEWP hydrolysates of PEP presented significantly higher reducing power than BA and NAT. In antimicrobial activities, *Escherichia coli*, *Salmonella typhimurium*, and *Pseudomonas aeruginosa* were not effectively inhibited by any DDEWP hydrolysates of PEP except for *Staphylococcus aureus*. Especially, the excellent antibacterial activity against *S. aureus* only was displayed in DDEWP hydrolysates of PEP 0.1%.

**Conclusion:**

DDEWP hydrolysates from PEP demonstrated significantly better DH, yield, DPPH radical scavenging activity and reducing power, furthermore, had excellent inhibitory on *S. aureus* due to large clear zone and moderated inhibitory in bactericidal inhibition.

## INTRODUCTION

Salted duck egg is one of the most popular preserved egg products and widely consumed in China and other parts of South-East Asia. However, salted duck egg white (SDEW) is normally discarded as waste product after collection of egg yolk and it may cause environmental pollution from high NaCl content (4% to 7%) [1]. In Taiwan, around 3,260 tons/yr are discarded [2].

Proteases are digestive enzymes that hydrolyze proteins to short peptides or free amino acids. They can generaly be divided into acid, neutral, and alkaline proteases. Proteases can be produced by microorganisms through fermentation or extracted from animal or plants tissues [3]. Protein hydrolysates have attracted increasing interests because of their safety and potential to act as multifunctional antioxidants to inhibit a variety of food ingredient oxidation pathways, including inactivation of scavenging by free radical, reactive oxygen species, reduction of hydroperoxide and chelation of pro-oxidative transition metals [4]. Pepsin is a gastric proteinase that is produced in the stomachs of vertebrates [5]. It has a broad specificity for peptide bonds, but preferentially cleaves bonds involving carbonyl groups of aromatic amino acids such as tryptophan, tyrosine, phenylalanine, leucine, and isoleucine, thereby producing small peptides [6]. *Bacillus* spp. including *B. licheniformis*, *B. subtilis*, *B. amyloliquefaciens*, and *B. majovenisi* are the most popular source of commercial alkaline proteases [7]. These proteases are broadly used in different industries such as leather, food, pharmaceutical, detergent, protein processing and peptide synthesis [8]. Nattokinase is an alkaline enzyme that isolated from the sticky component of natto or a cheese-like food made of soybeans fermented with *B. subtilis*. Nattokinase cleaves cross-linked fibrin and it possesses fibrinolytic/antithrombotic activity, antihypertensive, antiplatelet, anti-atherosclerotic and neuroprotective actions [9].

Recently, food protein hydrolysates have attached interest as a natural antioxidant and antimicrobial peptide, but until now there are only few studies characterizing antioxidant and antimicrobial activity of desalted duck egg white powder (DDEWP).

## MATERIALS AND METHODS

### Preparation of salted duck egg white

Cooled SDEW was purchased from Horn Liang Food Ltd. (Pingtung, Taiwan) and was transported to the laboratory of National Chung Hsing University (Taichung, Taiwan). Then, SDEW was stored at −20°C until use.

### Preparation of desalted duck egg white and desalted duck egg white powder

Frozen SDEW was thawed at 4°C overnight before homogenized at 8,000 rpm for 1 min and filtered through a 1 mm diameter sieve. SDEW was desalted using ultrafiltration desalinized technique (Easy-Pak Labuf-1812-1L, Lasers Technology, Taipei, Taiwan) according to the method of Fu [2]. SDEW was mixed with the same volume of RO water and desalinized by ultrafiltration technique then final desalted duck egg white (DDEW) was obtained with the salt content of less than 1%. Salt content was tested by using salt meter (Atago ES-421, Saitama, Japan). DDEW was spray dried by dry machine (B-191, BUCHI, Flawil, Switzerland) according to the method of Fu [2]. The conditions were: the inlet air temperature at 140°C, spray nozzle air speed at 10 L/min, the feed rate at 5 mL/min and the outlet temperature at 72°C. DDEWP was collected and stored in moisture proof cabinets (BK236, Bossmen, Taoyuan, Taiwan) at the relative humidity of 30% until use.

### Preparation of hydrolysis

Porcine pepsin (PEP, 601 U/mg) was purchased from Sigma-Aldrich (St. Louis, MO, USA), promod 223P-P223P proteinase from *Bacillus* spp. (BA, 90 U/g) received from Biocatalysis Ltd. (Wales, UK), and nattokinase from *B. subtilis* var. natto (NAT, 20,000 FU/g) obtained from Greenyn Biotechnology (Taichung, Taiwan). DDEWP was dissolved in 100 mL reverse osmosis (RO) water at a ratio of 1:6 (w/v). Then the enzymatic hydrolysis was performed by using PEP, BA, and NAT with ratio of enzyme to substrate 0.1%, 0.3%, and 0.5% (w/v) at their optimal conditions: PEP (pH 2.5, 37°C), BA and NAT (pH 6.5, 37°C) for 1, 3, 6, 9, 12, and 24 h, respectively. The pH of the mixture was maintained constant during hydrolysis using 0.5 M NaOH and 0.5 M HCl. Hydrolysis was stopped by heating at 95°C for 10 min to inactivate the enzyme.

### Determination of the degree of hydrolysis

The degree of hydrolysis (DH) was measured using 2,4,6-Trinitrobenzenesulfonic acid solution (TNBS) method referred by Hsu [10] with modifications. Hydrolysate sample (125 μL) with an appropriated concentration was mixed with 2 mL of 0.2125 M phosphate buffer (pH 8.2) and 1 mL of TNBS (Sigma-Aldrich, USA). After incubation in the dark at room temperature for 30 min, 2 mL of 0.1 M Na_2_SO_3_ was added to terminate the reaction. The mixture was cooled for 15 min in running water. Absorbance at 420 nm was measured using a spectrophotometer (U3210, Hitachi, Tokyo, Japan). An ascending concentration series of *L*-leucine (Leu) was used as a standard curve of α-amino acid content. DH was calculated as follows:

(1) $$\%\text{DH}=\left[\frac{\left(\text{Lt}-\text{L}0\right)}{\left(\text{Lmax}-\text{L}0\right)}\right]\times 100$$

where L_t_ is the amount of α-amino acid released at time t. L_0_ is the amount of α-amino acid in the original egg white solution. L_max_ is the total α-amino acid in original egg white solution obtained after acid hydrolysis with 6 N HCl at 105°C for 24 h.

### Determination of yield

Hydrolysate sample was centrifuged at 8,000 rpm for 20 min at 4°C. The supernatant was collected, freeze-dried by lyophilizer (Kingmech FD12-12PL, Taoyuan, Taiwan) and stored in moisture proof cabinets until used. The yield of the sample was calculate using the following equation:

(2) $$\text{Yield }(\%)=\frac{\text{weight of lyophilized supernatant sample}}{\text{weight of initial desalted egg white powder sample}}\times 100$$

### Determination of 2, 2 –diphenyl-1-picrylhydrazyl radical scavenging activity

The 2, 2 –diphenyl-1-picrylhydrazyl (DPPH) radical scavenging activity of the hydrolysate was determined according to Morales-Medina et al [11] with some modifications. The lyophilized sample was dissolved with RO water (1.25 mg/mL). Sample at 2.5 mL was mixed with DPPH methanolic solution (0.3 mM, 1 mL). The mixture was kept at room temperature in the dark for 30 min, and the absorbance was measured at 517 nm with a spectrophotometer. The control was conducted in the same method, except that methanol was used instead of sample. Butylated hydroxytoluene (BHT) 200 mg/L was used as a positive control. The DPPH radical scavenging activity was calculated as follows:

(3) $$\text{Scavenging activity }(\%)=\left[\frac{1-(\text{As}-\text{Ab})}{\text{Ac}}\right]\times 100$$

where A_c_ is the absorbance of the control reaction, A_s_ is the absorbance of the sample and A_b_ is the absorbance of the sample without DPPH. Values presented are the mean of triplicate analyses.

### Ferrous ion chelating ability assay

The ferrous ion chelating ability test was carried out according to Yu and Tan [12]. In the dark, 1 mL sample solution was mixed with 3.7 mL of methanol and then allowed to react with 100 μL of 2 mM FeCl_2_·4H_2_O solution for 30 s. The RO water was used instead of sample solution in the control. The RO water was used instead of reagent solution as a blank. The reaction was initiated by mixing 200 μL of 5 mM ferrozine. After 10 min of incubation at room temperature, the absorbance was measured at 562 nm. Ethylenediaminetetraacetic acid (EDTA) 100 mg/L was used as a positive control. The results were calculated according to the following formula:

(4) $$\text{Chelating ability }(\%)=\left[\frac{1-(\text{As}-\text{Ab})}{\text{Ac}}\right]\times 100$$

where A_s_ is the absorbance of the sample, A_b_ is the absorbance of the blank and A_c_ is the absorbance of the control. Values presented the mean of triplicate analyses.

### Reducing power assay

The reducing power of the hydrolysate was measured using a modified method of Yu and Tan [12] with some modifications. One mL of protein hydrolysate, 2.5 mL of 0.2 M phosphate buffer (pH 6.6) and 2.5 mL of 1% (w/v) K_3_Fe(CN)_6_ solution were vigorously mixed in test tube. After 30 min incubation at 50°C, 2.5 mL of 10% (w/v) trichloroacetic acid was added and the reaction mixture was centrifuged at 10,000 ×*g* for 10 min. Then, 500 μL of the supernatant was mixed with 4 mL of RO water and 0.25 mL of 0.1% (w/v) FeCl_3_. After 10 min of incubation at room temperature, the absorbance of the supernatant was determined at 700 nm. BHT 200 mg/L was used as a positive control. An increased absorbance of the reaction mixture indicated the increased reducing power. Values presented are the mean of triplicate analyses.

### Bacterial strains and cultivation conditions

*Staphylococcus aureus* (BCRC 11863), *Escherichia coli* (BCRC 11509), *Salmonella typhimurium* (BCRC 10905), and *Pseudomonas aeruginosa* (BCRC 11864) were supplied from the Department of Veterinary Science of National Chung Hsing University (Taichung, Taiwan). Bacterial strains were separately cultured in tryptic soy agar (TSA) (Merk, Darmstadt, Germany) at 37°C. All stock cultures were maintained at −65°C in 20% (v/v) glycerol as a cryoprotectant. During the experiments, all strains were sub-cultured every 2 weeks on agar media.

### Determination of antibacterial activity

Antibacterial activity assay using agar diffusion method was performed according to the method of Jemil et al [13]. The protein hydrolysates were dissolved in sterilized water at a concentration of 150 mg/mL. Sterilized TSA agar (18 mL) was distributed into petri dish. After solidification, TSA agar was drilled with a sterile tube (1.1 cm diameter) under aseptic conditions. Then, 200 μL of test bacterial strain (10^6^ colony-forming unit/mL) were spread on the agar, subsequently wells were filled with 200 μL of sample. DDEWP dissolved in sterilized water at a concentration of 150 mg/mL was used as control. Plates were incubated at 37°C for 18 h. Ampicillin (10 μg/disc) (Oxoid, London, UK) was used as positive referent standards to determine the sensitivity of *E. coli*, *S. typhimurium*, and *S. aureus*. Ceftazidime (30 μg/disc) (Oxiod, London, UK) was used as positive control for *P. aeruginosa*. Diameters of the inhibition zones (DIZ) were measured in centimeters, minus the well diameter. The formation of DIZ areas are referred: ≥1.5 cm diameters around the wells were regarded as strongly inhibitory against the bacteria, 1.0 to 1.5 cm zone of inhibition is moderately inhibitory and 0.6 to 1.0 cm zone of inhibition is mild inhibitory and <0.6 cm zone of inhibition is non-inhibitory.

### Statistical analysis

Data were analyzed using Statistical Analysis System’s Procedures (SAS Institute Inc., Cary, NC, version 9.4, 2017) with a 5% level of significance. Analysis of variance was performed to analyze the effect of treatment. Means comparisons were separated using Tukey’s new multiple range test. The correlations analysis was performed using the Pearson correlation coefficient. All analyses were conducted in triplicate and averaged.

## RESULTS AND DISCUSSION

### Degree of hydrolysis and yield

**Figure 1:**
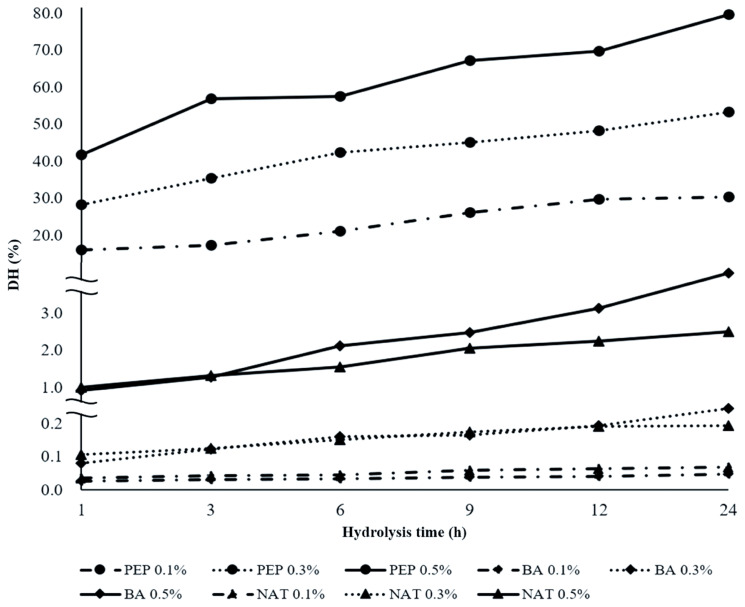
Degree of hydrolysis (DH) of desalted duck egg white powder (DDEWP) hydrolyzed by different enzyme types, concentrations and times. PEP 0.1%, pepsin 0.1%; PEP 0.3, pepsin 0.3%; PEP 0.5%, pepsin 0.5%; BA 0.1%, *Bacillus* spp. proteinase 0.1%; BA 0.3%, *Bacillus* spp. proteinase 0.3%; BA 0.5%, *Bacillus* spp. proteinase 0.5%; NAT 0.1%, Nattokinase 0.1%; NAT 0.3%, Nattokinase 0.3%; NAT 0.5%, Nattokinase 0.5%.

The data of DH of DDEWP which was hydrolyzed by different enzymes and ratio of enzyme to substrate at optimal incubated condition are shown in Figure 1. All PEP treatments exhibited the most effective hydrolysis at the same ratio of enzyme to substrate and similar incubation times compared with BA and NAT. The DH of PEP 0.5% obtained 56.86% after 3 h of hydrolysis and which increased with time. Finally, the highest DH of 79.59% was recorded at the end of 24 h of hydrolysis. In addition, the higher ratio of enzyme to substrate and longer time, the higher degree hydrolysis was found in pepsin treatment. However, both of BA and NAT showed substantially lower DH values and did not exceed 5% by the end of hydrolysis. In this study, NAT was almost inactive against the substrate during the whole hydrolysis process, regardless of ratio of enzyme to substrate and time. These results indicated DDEWP was suitable as substrate for PEP but was not for NAT and BA. The study of Ruan et al [14] conducted that at the optimal conditions for pepsin hydrolysis (enzyme 0.80 g/L, substrate 105 g/L, pH 2.0, 45°C and 180 min), allowed the DH to reach the highest values.

In this study, the yield was dependent on enzyme type and concentrate. The yield of all treatments are presented in Table 1. The yield of all treatments increased with the increase of enzyme concentration and hydrolysis time. Based on the analysis of Table 1, each PEP treatment obtained the highest yield and significantly higher than that of BA and NAT at each concentration and hydrolysis time. The yield after 24 h was 14.7% in 0.5% PEP, 6.47% in 0.5% BA and 6.76% in 0.5% NAT, separately. These results also indicated that DDEWP could be a suitable substrate for PEP compared with BA and NAT. Barać et al [15] clarified that several factors had an effect on the protein hydrolysates such as type of enzyme, treatment conditions including enzyme/substrate ratio, temperature, time and type of substrate. Ruan et al [14] also stated that the sufficient of substrate and enzyme concentration could increase the enzymatic reaction rate, which directly affected yield rate.

As evidenced in Table 2, there was a positive significant correlation (p<0.05) between DH and yield of peptic hydrolysates (*r* = 0.97). In this study, the yield of all treatments increased with DH, especially, 0.5% PEP obtained the highest DH value and yield compared to BA and NAT.

### 2, 2–Diphenyl-1-picrylhydrazyl radical scavenging activity

**Figure 2:**
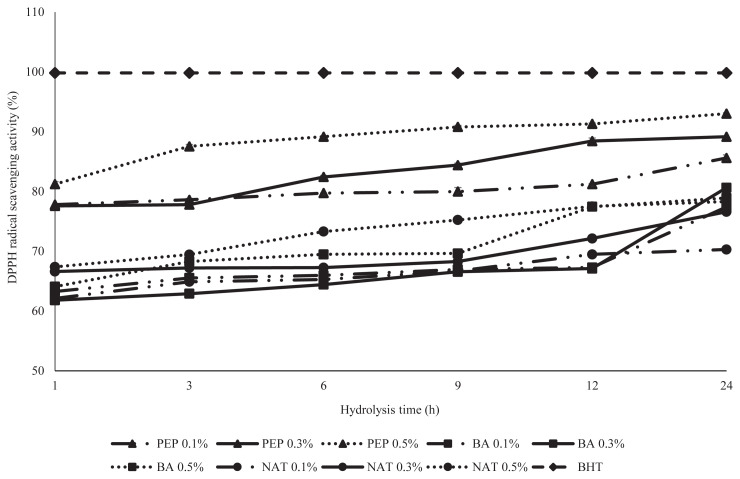
Analysis of 2, 2–diphenyl-1-picrylhydrazyl (DPPH) free radical of desalted duck egg white powder (DDEWP) hydrolysates prepared by different enzyme types, concentrations and hydrolysis times. PEP 0.1%, pepsin 0.1%; PEP 0.3, pepsin 0.3%; PEP 0.5%, pepsin 0.5%; BA 0.1%, *Bacillus* spp. proteinase 0.1%; BA 0.3%, *Bacillus* spp. proteinase 0.3%; BA 0.5%, *Bacillus* spp. proteinase 0.5%; NAT 0.1%, Nattokinase 0.1%; NAT 0.3%, Nattokinase 0.3%; NAT 0.5%, Nattokinase 0.5%.

DPPH is a stable free radical that is widely used to determine the capacity of an antioxidant, when it accepts an electron or hydrogen radical the change of color is correlated with the sample antioxidant activity [16]. The DPPH radical scavenging activity of hydrolysates from PEP, BA, and NAT are demonstrated in Figure 2. The DPPH activities of DDEWP hydrolysates were influenced by the time of hydrolysis and the type of enzyme. Among the different hydrolysates, PEP exhibited significantly higher DPPH radical scavenging activity than BA and NAT. In the analysis of data, all hydrolysate of PEP displayed more than 85.6% radical scavenging activity, and less than 75% and 70% for BA and NAT, respectively. This result was agreed with He et al [17] who showed that rapeseed protein hydrolysis with pepsin + pancreatin had significantly the highest scavenging activity against DPPH radical than proteinase, alcalase and flavozyme. Additionally, Sun et al [18] also reported that peptic hydrolysate of porcine hemoglobin exhibited the highest scavenging activity compared to other hydrolysates derived from various proteases. Overall, Je et al [19] reviewed peptic hydrolysate and showed that it had the highest antioxidant activity (DPPH radical scavenging) compared to hydrolysates produced by other commercial enzymes (alcalase, chymotrypsin, neutrase, papain, and trypsin) in tuna backbone. However, all treatments of BA and NAT had less than 75% DPPH radical scavenging activity compared with PEP and probably resulted from a small value in DH of both enzymatic treatments. Liu et al [20] suggested that the DPPH radical scavenging activity increases with an increase in the DH due to the correlation between the DH and the antioxidant activity of protein hydrolysates. Sbroggio et al [21] mentioned that the antioxidant capacity of protein hydrolysates are influenced by the degree of the hydrolysates and type of enzyme. Actually, a higher correlation coefficient (0.88) was also found between DH and DPPH radical scavenging activity of DDEWP hydrolysates (Table 2) in this study.

### Ferrous ion chelating activity

**Figure 3:**
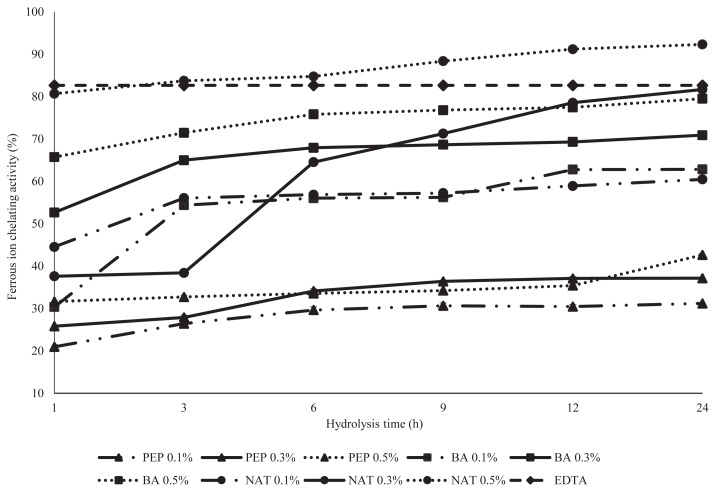
Analysis of ferrous ion chelating activity of desalted duck egg white powder (DDEWP) hydrolysates prepared by different enzyme types, concentrations and hydrolysis times. PEP 0.1%, pepsin 0.1%; PEP 0.3%, pepsin 0.3%; PEP 0.5%, pepsin 0.5%; BA 0.1%, *Bacillus* spp. proteinase 0.1%; BA 0.3%, *Bacillus* spp. proteinase 0.3%; BA 0.5%, *Bacillus* spp. proteinase 0.5%; NAT 0.1%, Nattokinase 0.1%; NAT 0.3%, Nattokinase 0.3%; NAT 0.5%, Nattokinase 0.5%.

Transition metals such as Fe^2+^ and Cu^2+^ are also an *in vitro* method for the measurement of protein hydrolysates in metal chelating activity. Transition metal ions are able catalyze the generation of reactive of oxygen species such as hydroxyl radical (OH^•^) and superoxide anion (O_2_^−^) [22]. The disruption of the formation of the complex Fe^2+^ ferrozine indicated by the decrease of the purple color development was used to determine the chelating capacity of DDEWP hydrolysates by PEP, NAT, and BA in this experiment (Figure 3). In this study, a significantly converse result of ferrous ion chelating activity occurred in all PEP treatments compared with that of NAT and BA. All DDEWP hydrolysates from PEP had lower ferrous ion chelating activity (<37%) and significantly lower than that of NAT (>37%–92%) and BA (30% to 79%). The results also indicated that the same DDEWP enzymatic hydrolysate displayed significantly different antioxidant activity in DDPH and ferrous ion chelating activity. Therefore, the tendency may be used as a good indicator for antioxidative evaluation in different food systems in the future. Besides, both DDEWP hydrolysates from 0.5% BA and NAT at 24 h exhibited greater ferrous ion chelating activity than that of PEP. Especially, DDEWP hydrolysates from 0.5% NAT at 24 h possessed 92.32% ferrous ion chelating activity and even significantly higher than the positive control-EDTA (82.67%) in this experiment. This result was probably due to low DH and resulted in longer peptide chains in BA and NAT which then formed strong complexes with metal ions. Many similar conclusions also were reported by Shi et al [23] who demonstrated that the synergistic effects of long chain peptide residues had an increased chelating activity compared to shorter peptides. Intarasirisawat et al [24] also stated that peptide chain length was an important factor for metal chelation of skipjack roe protein hydrolysates. Besides, Tang et al [25] found that the long-chain peptide of hemp protein hydrolysates exhibited higher chelating activity than those of short-chain peptide hydrolysates. Moreover, He et al [17] revealed that unfractionated rapeseed protein hydrolysates and high molecular weight (5 to 10 kDa) peptide fractions showed a higher chelating capacity than did low molecular weight peptide fractions (<3 kDa). In this study, there was a clear negative correlation (*r* = −0.63) between the DH and the chelating activity of DDEWP hydrolysate, and means that hydrolysates with high DH value presented low chelating activity.

### Reducing power

**Figure 4:**
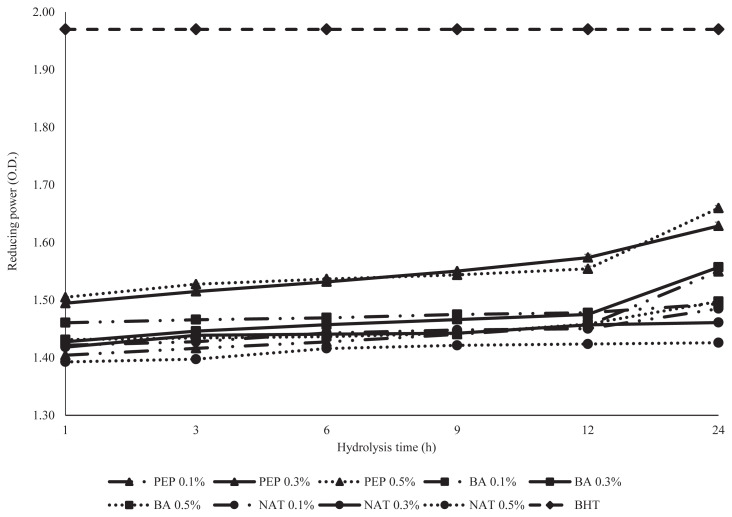
Analysis of reducing power of desalted duck egg white powder (DDEWP) hydrolysates prepared by different enzyme types, concentrations and hydrolysis times. PEP 0.1%, pepsin 0.1%; PEP 0.3%, pepsin 0.3%; PEP 0.5%, pepsin 0.5%; BA 0.1%, *Bacillus* spp. proteinase 0.1%; BA 0.3%, *Bacillus* spp. proteinase 0.3%; BA 0.5%, *Bacillus* spp. proteinase 0.5%; NAT 0.1%, Nattokinase 0.1%; NAT 0.3%, Nattokinase 0.3%; NAT 0.5%, Nattokinase 0.5%.

Reducing power is widely used as one of the antioxidant capability indicators of protein hydrolysates. The Fe^2+^ formed was monitored by measuring the formation of Perl’s Prussian blue at 700 nm. Samples with a higher reducing power have enhanced capacities to donate electrons [25]. The ability of a sample to reduce Fe^3+^ complex to Fe^2+^ form is shown in Figure 4. DDEWP hydrolysates of PEP presented significantly higher reducing power than BA and NAT. The highest reducing power was 1.51 to 1.66 in the PEP 0.5% enzyme concentration. All PEP groups showed high reducing power, while BA and NAT exhibited low reducing power. However, the lowest reducing power was less than 1.4 and found in 0.5% NAT. Overall, there was a clear positive correlation between the DH and the reducing power of the hydrolysates (Table 2), reducing power of DDEWP hydrolysates increased with increasing DH values, which may be due to a direct relationship between the DH of hydrolysates and the capacity of hydrolysates to donate their electrons to free radicals. Similar results were also found in discarded Mediterranean fish [26], and yellow strip trevally protein hydrolysates [27].

### Antibacterial activity

The antibacterial activity of all enzymatic hydrolysates were evaluated against four selected bacteria such as *S. aureus*, *E. coli*, *S. typhimurium*, and *P. aeruginosa* in this study. The antibacterial activity was assessed by evaluating the inhibition zones (DIZ). Unfortunately, no inhibition zones for four selected bacteria were found in hydrolysates from NAT and BA at different concentrations and hydrolysis time in this research. The results might be due to a very low DH value in both enzymes at hydrolysis period (Figure 1). In addition, antibacterial activities of all DDEWP hydrolysates of PEP are shown in Table 3 and 4. Analysis of data from Table 3 and 4 indicates that *E. coli*, *S. typhimurium*, and *P. aeruginosa* were not effectively inhibited by all DDEWP hydrolysates of PEP while *S. aureus* was inhibited. The antibacterial activity against *S. aureus* only was displayed in DDEWP hydrolysates of PEP 0.1% and decreased with the increase of PEP concentration in this experiment. In more detail, DDEWP hydrolysates with PEP 0.1% at 6, 9, and 12 h hydrolysis demonstrated the most efficient bactericidal inhibition and displayed the largest clear zone at 1.03 cm and moderated inhibitory (++). The main cause might be that the DH value of PEP 0.1% was lower than PEP 0.3% and 0.5% and enzyme cleavage sites may have produced different peptide fractions. These results were a clear negative correlation (p<0.05) between the DH and the antimicrobial activity of peptic DDEWP hydrolysate (Table 2). Ghanbari et al [28] suggested that amino acid sequence, secondary structure, length, molecular weight and charge had affected on the antimicrobial activity of a peptide. Tejano et al [29] also demonstrated that higher DH did not necessarily correlate to higher bioactivity. Many antimicrobial peptides derived from enzyme hydrolysis of egg white have been identified in previous studies. Lin et al [30] reported that hydrolysates of egg white possessed antioxidant and/or antimicrobial effects.

### Correlation between degree of hydrolysis, yield, antimicrobial and antioxidant activities

The correlation (*r*) among DH, yield, antimicrobial and antioxidant activities of peptic DDEWP hydrolysates was analyzed by Pearson correlation analysis. The results are presented in Table 2 and show that DH had a significant positive correlation (p<0.05) with the yield, DPPH radical scavenging activity and reducing power; the coefficients r were 0.97, 0.88, and 0.76, respectively. However, DH had a significant negative correlation (p<0.5) between with Fe^2+^-chelating ability and coefficient *r* was −0.63. These results indicated that the DH played an important role in the yield and antioxidant activities of peptic DDEWP hydrolysates. In addition, the correlation between DH and antimicrobial activity of peptic DDEWP hydrolysates is shown in Table 2. Statistically significantly negative correlation (p<0.05) was found between DH and antimicrobial activity of inhibiting *E. coli* and *P. aeruginosa*. The negative correlation between these parameters was *r* = −0.72 and −0.45, respectively. However, no significant correlation (p>0.5) was found between DH and the antimicrobial activity of *S. aureus* and *S. typhimurium* (*r* = −0.21 and *r* = −0.10, respectively).

## CONCLUSION

In conclusion we found that DDEWP hydrolysates from pepsin had not only a significantly better DH, yield, DPPH radical scavenging activity and reducing power, but also had excellent inhibition against *S. aureus* compared with those of BA and NAT. However, among peptic DDEWP hydrolysates, 0.1% PEP possessed the most effective antibacterial activity on *S. aureus* in this experiment. Thus, peptic DDEWP hydrolysates can be applied as a natural antioxidative and antimicrobial substance in food ingredients in food processing.

## Figures and Tables

**Table 1:** Yield of desalted duck egg white hydrolysates prepared by different enzyme types, concentrations and hydrolysis times

Enzyme1)	Hydrolysis time (h)					

	1	3	6	9	12	24
PEP 0.1%	8.76±1.27^C^^a^	8.24±0.63^C^^a^	9.29±0.91^B^^a^	9.89±0.67^B^^a^	10.24±0.49^B^^a^	10.31±0.77^B^^a^
PEP 0.3%	10.93±0.64^B^^c^	11.38±0.73^B^^bc^	12.42±1.35^A^abc	13.07±0.66^A^abc	13.51±1.02^A^^ab^	14.04±0.38^A^^a^
PEP 0.5%	12.87±0.12^A^^a^	13.44±1.32^A^^a^	13.56±0.71^A^^a^	13.82±0.97^A^^a^	14.40±1.29^A^^a^	14.71±1.25^A^^a^
BA 0.1%	3.47±0.24^E^^b^	3.73±0.76^E^^ab^	4.33±0.53^D^^ab^	4.33±0.84^D^^ab^	4.98±0.57^C^^ab^	5.13±0.27^C^^a^
BA 0.3%	4.58±0.23^DE^^a^	4.82±0.88^DE^^a^	4.96±0.10^CD^^a^	5.02±0.73^CD^^a^	5.13±0.33^C^^a^	5.36±0.10^C^^a^
BA 0.5%	5.29±0.20^D^^a^	5.73±0.52^DE^^a^	5.71±0.10^CD^^a^	6.13±0.59^CD^^a^	6.11±0.71^C^^a^	6.47±0.24^C^^a^
NAT 0.1%	4.27±0.58^DE^^a^	4.71±0.33^DE^^a^	4.89±0.23^CD^^a^	4.96±0.57^CD^^a^	4.98±0.53^C^^a^	5.02±0.89^C^^a^
NAT 0.3%	5.02±0.32^DE^^a^	5.22±0.15^DE^^a^	5.53±0.48^CD^^a^	5.60±0.33^CD^^a^	5.53±0.70^C^^a^	5.67±0.35^C^^a^
NAT 0.5%	5.47±0.40^D^^a^	5.98±0.53^D^^a^	6.42±0.81^C^^a^	6.60±0.18^C^^a^	6.60±0.68^C^^a^	6.76±0.63^C^^a^

Mean±standard error (n = 3).

1) PEP 0.1%, pepsin 0.1%; PEP 0.3%, pepsin 0.3%; PEP 0.5%, pepsin 0.5%; BA 0.1%, *Bacillus* spp. proteinase 0.1%; BA 0.3%, *Bacillus* spp. proteinase 0.3%; BA 0.5%, *Bacillus* spp. proteinase 0.5%; NAT 0.1%, Nattokinase 0.1%; NAT 0.3%, Nattokinase 0.3%; NAT 0.5%, Nattokinase 0.5%.

A–E The letter in the same column mean there are significant differences (p<0.05) between different samples at the same hydrolysis times.

a–c The different letters in the same row indicate that there are significant differences (p<0.05) for the same sample at different hydrolysis times.

**Table 2:** Pearson correlations of degree of hydrolysis, yield, antibacterial and antioxidant activity of desalted duck egg white powder hydrolysates

Item	DH	Yield
DH	1	-
Yield	0.97***	1
DPPH radical scavenging activity	0.88***	0.90***
Fe^2+^-chelating activity	−0.63***	−0.60***
Reducing power	0.76***	0.71***
*S. aureus*	−0.21	-
*E. coli*	−0.72**	-
*S. typhimurium*	−0.10	-
*P. aeruginosa*	−0.45*	-

DH, degree of hydrolysis; DPPH, 2, 2–diphenyl-1-picrylhydrazyl.

* p<0.05,

** p<0.01,

*** p<0.001.

**Table 3:** Analysis of inhibition zone of selected bacteria treated with desalted duck egg white powder hydrolysates by pepsin at different concentrations and hydrolysis times

Bacteria kind	Enzyme level1)	Hydrolysis time (h)						Ampicillin	Ceftazidime

		1	3	6	9	12	24		
		------------------------------------------------------- Diameter of inhibition zone (cm)2) -------------------------------------------------------------							
*S. aureus*	PEP 0.1%	0.87±0.03^A^^b^	0.90±0.01^A^^b^	1.03±0.03^A^^a^	1.03±0.03^A^^a^	1.03±0.01^A^^a^	0.90±0.01^A^^b^	1.93±0.06	-
	PEP 0.3%	0.77±0.03^B^^c^	0.80±0.01^B^^c^	0.97±0.03^B^^a^	0.95±0.05^B^^a^	0.97±0.03^B^^a^	0.87±0.02^A^^b^		
	PEP 0.5%	0.67±0.03^C^^c^	0.86±0.02^C^^ab^	0.89±0.01^C^^a^	0.90±0.01^C^^a^	0.90±0.01^C^^a^	0.83±0.03^B^^b^		
*E. coli*	PEP 0.1%	0.22±0.02^A^^a^	0.26±0.03^A^^a^	0.25±0.01^A^^a^	0.26±0.01^A^^a^	0.25±0.03^A^^a^	0.24±0.02^A^^a^	1.70±0.10	-
	PEP 0.3%	0.16±0.01^B^^a^	0.19±0.03^AB^^a^	0.19±0.03^B^^a^	0.20±0.02^B^^a^	0.20±0.02^AB^^a^	0.19±0.01^B^^a^		
	PEP 0.5%	0.14±0.01^B^^a^	0.14±0.02^B^^a^	0.13±0.03^C^^a^	0.17±0.03^B^^a^	0.17±0.03^B^^a^	0.15±0.01^C^^a^		
*S. typhimurium*	PEP 0.1%	0.27±0.01^A^^b^	0.27±0.01^A^^b^	0.30±0.01^A^^b^	0.43±0.06^A^^a^	0.40±0.01^A^^a^	0.40±0.01^A^^a^	2.23±0.06	-
	PEP 0.3%	0.17±0.03^B^^c^	0.23±0.03^A^^b^	0.23±0.03^B^^b^	0.34±0.01^B^^a^	0.34±0.01^B^^a^	0.29±0.01^B^^ab^		
	PEP 0.5%	0.23±0.02^A^^a^	0.27±0.03^A^^ab^	0.27±0.05^AB^^ab^	0.29±0.01^B^^a^	0.29±0.01^C^^a^	0.29±0.01^B^^a^		
*P. aeruginosa*	PEP 0.1%	0.37±0.03^A^^c^	0.41±0.01^A^^bc^	0.41±0.02^A^^b^	0.50±0.01^A^^a^	0.49±0.01^A^^a^	0.41±0.01^A^^b^	-	1.37±0.06
	PEP 0.3%	0.23±0.01^B^^e^	0.26±0.01^B^^d^	0.30±0.01^B^^c^	0.40±0.01^B^^a^	0.40±0.01^B^^a^	0.37±0.02^A^^b^		
	PEP 0.5%	0.21±0.02^B^^b^	0.26±0.04^B^^ab^	0.30±0.01^B^^a^	0.31±0.01^C^^a^	0.31±0.02^C^^a^	0.27±0.03^B^^ab^		

Mean±standard error (n = 3).

1) PEP 0.1%, pepsin 0.1%; PEP 0.3%, pepsin 0.3%; PEP 0.5%, pepsin 0.5%.

2) Diameter of inhibition zone (cm), which minus with the well diameter of 1.1 cm or positive disc diameter of 0.6 cm.

A–C The letter in the same column mean there are significant differences (p<0.05) between different samples at the same hydrolysis times.

a–e The different letters in the same row indicate that there are significant differences (p<0.05) for the same sample at different hydrolysis times.

**Table 4:** The scale of measurement of selected bacteria treated with desalted duck egg white powder hydrolysates by pepsin at different concentrations and hydrolysis times

Bacteria	Enzyme1)	Time of hydrolysis (h)2)						Ampicillin	Ceftazidime

		1	3	6	9	12	24		
*S. aureus*	PEP 0.1%	+	++	++	++	++	+	+++	−
	PEP 0.3%	+	+	+	+	+	+		
	PEP 0.5%	+	+	+	+	+	+		
*E. coli*	PEP 0.1%	−	−	−	−	−	−	+++	−
	PEP 0.3%	−	−	−	−	−	−		
	PEP 0.5%	−	−	−	−	−	−		
*S. typhimurium*	PEP 0.1%	−	−	−	−	−	−	+++	−
	PEP 0.3%	−	−	−	−	−	−		
	PEP 0.5%	−	−	−	−	−	−		
*P. aeruginosa*	PEP 0.1%	−	−	−	−	−	−	−	+++
	PEP 0.3%	−	−	−	−	−	−		
	PEP 0.5%	−	−	−	−	−	−		

1) PEP 0.1%, pepsin 0.1%; PEP 0.3%, pepsin 0.3%; PEP 0.5%, pepsin 0.5%.

2) The scale of measurement was the following: +++strong inhibitory (≥1.5 cm), ++ moderated inhibitory (1.0 to 1.5 cm), + mild inhibitory (0.6 to 1.0 cm), − no inhibitory (<0.6 cm).
